# Impact of Measures Aiming to Reduce Sugars Intake in the General Population and Their Implementation in Europe: A Scoping Review

**DOI:** 10.3389/ijph.2021.1604108

**Published:** 2022-01-13

**Authors:** Sophie Bucher Della Torre, Clémence Moullet, Corinne Jotterand Chaparro

**Affiliations:** Department of Nutrition and Dietetics, Geneva School of Health Sciences, HES-SO University of Applied Sciences and Arts Western Switzerland, Geneva, Switzerland

**Keywords:** taxation, sugars, sugar-sweetened beverages, food environments, nutrition education, population interventions, scoping review

## Abstract

**Objectives:** Implementing public health measures is necessary to decrease sugars intake, which is associated with increased risk of noncommunicable diseases. Our scoping review aimed to identify the types of measures implemented and evaluated to decrease sugars intake in the population and to assess their impact.

**Methods:** Following a review of systematic reviews (SRs) published in 2018, we systematically searched new SR (May 2017–October 2020) in electronic databases. We also searched the measures implemented in Europe in the NOURISHING database. Two researchers selected the reviews, extracted and analysed the data.

**Results:** We included 15 SRs assessing economic tools (n = 5), product reformulation and labels/claims (n = 5), and educational/environmental interventions (n = 7). Economic tools, product reformulation and environmental measures were effective to reduce sugar intake or weight outcomes, while labels, education and interventions combining educational and environmental measures found mixed effects. The most frequently implemented measures in Europe were public awareness, nutritional education, and labels.

**Conclusion:** Among measures to reduce sugar intake in the population, economic tools, product reformulation, and environmental interventions were the most effective, but not the more frequently implemented in Europe.

## Introduction

The frequent consumption of excessive dietary sugars, especially sugar-sweetened beverages (SSBs) is a risk factor for unbalanced diet, weight gain, and an increased risk of noncommunicable diseases including type 2 diabetes [1, 2], cardiovascular disease mortality [3], and dental caries [4, 5]. A causal link between a high-sugar diet and obesity has been found and explained by free sugars, especially in liquid form [5, 6]. According to the World Health Organization (WHO), the term “free sugars” includes all “monosaccharides and disaccharides added to foods by the manufacturer, cook or consumer, and sugars naturally present in honey, syrups, fruit juices and fruit juice concentrates” [5].

The WHO strongly recommend that intake of free sugars should not exceed 10% of total energy intake, and a conditional recommendation states that it should not exceed 5% [5]. This represents a maximum intake of 50 g/day, ideally 25 g/day, for a person consuming 2,000 calories. In a review comparing total sugars (naturally present and added in food and drink) in several European countries, the mean intake ranged from 76 g/day in Spain to 117 g/day in the Netherlands [7]. In a Swiss survey, the mean intake of total sugars reached 107 g/day (11% of total energy intake), and only 8% of the population followed the WHO recommendation of less than 5% of total energy intake [8].

Many scientific organizations and authorities at international, national and local levels have issued policy recommendations that aim to reduce sugars intake, with a focus on children given their inclination to have higher sugar intake [9]. These include a range of public health measures, such as consumer education, food and nutrition labelling, regulation of the marketing, fiscal policies, population intake monitoring and product reformulation [2]. Governments around the world have already adopted different measures to reduce sugar intake, and their level of implementation differs among the countries, as shown by the NOURISHING database, a tool developed by the World Cancer Research Fund International that indexes by country the measures designed to tackle unhealthy diets [10, 11].

In 2018, a review of reviews published by Kirkpatrick et al. assessed both the impact of measures aiming to decrease sugars intake among populations and the gaps in the available evidence [12]. Based on 12 systematic reviews (SRs) published between 2006 and 2016, the authors concluded that some interventions had the potential to reduce the intake of SSBs including taxes, modification of food environments, and, lastly health promotion and education. However, the limited available evidence and a high heterogeneity of methods and measures in included studies prevented the authors from drawing firm conclusions about the effectiveness of the interventions. Since 2016, numerous studies and reviews have significantly grown the body of evidence on this topic.

Considering the recent literature and the different approaches implemented by countries to reduce sugar intake in the population, it was important to conduct a scoping review that aimed to gather and analyse evidence on this topic, by assessing which measures have been scientifically evaluated, what were their impact, and which measured have already been implemented in Europe. This would help the decision-makers of European countries to identify which measures are preferable. The primary objective of our scoping review was to determine the types of measures studied to decrease sugars intake in the population and their impact. The secondary objective was to identify the measures implemented in Europe.

## Methods

### Type of Research Conducted

We conducted a scoping review in two steps. Firstly, we searched the SRs that assessed the impact of the measures implemented to reduce sugar intake in the population. The review of reviews of Kirkpatrick et al. also addressed this research question, based on SRs published until 2016. Thus, we focused on SRs published after this date and used a similar research methodology. The main findings of Kirkpatrick et al. were included in our analysis and compared to the more recent data. In addition, we searched the NOURISHING database in order to compile the measures that have been implemented by European countries [11].

Initially, our research group was commissioned by the Swiss Federal Food Safety and Veterinary Office FSVO to write a scientific report providing an overview of the strategic options for reducing sugar consumption in Switzerland at the population level [13]. This previous report used international scientific data, but focused on Switzerland. Therefore, the scoping of the current review was expanded to Europe.

A research protocol was developed before this scoping review was conducted, but it was not published. We planned initially to search SRs published until the end of 2019, however as new SRs were published, we extended the publication data and updated the search in October 2020. The PRISMA checklists can be found in Supplementary File S1.

### Search of SRs on the Impact of the Measures Implemented to Reduce Sugar Intake

#### Research Question

Our research question was as follows: “What is the impact of public health measures to reduce sugar consumption on sugar intake and health outcomes in the population?”.

#### Inclusion/Exclusion Criteria

Publications were eligible for inclusion in this review if they were SRs published in English, French, German, or Spanish. Kirkpatrick et al. searched SRs published in English from January 2005 to May 2017 [12]. Therefore, we searched for SRs published between May 2017 and October 2020. Reviews that did not report a systematic search strategy to identify the literature were excluded, but SRs that did not include a meta-analysis were eligible.

We considered the pediatric and adult populations. The interventions included all type of interventions that aimed to support reductions in sugar consumptions among populations, at various levels (e.g., regional, national, and global). These include different study designs such as randomized control trials, nonrandomized controlled trials, pre-post studies, modelling studies, laboratory studies, etc. When the intervention did not aim to decrease specifically sugar intake, i.e., a program to prevent obesity, the SR was excluded. The outcomes could be either sugar intake, SSB consumption and/or any health outcomes.

#### Search Strategies and Identification of SRs

The search strategy for Medline PubMed used by Kirkpatrick et al. was used to identify recent studies. The full search strategy, checked by an experienced librarian, including all identified keywords and MeSH terms, is presented in Supplementary File S2. In the Cochrane Database of Systematic Reviews, we used the general term “Sugar” and restricted the search to May 2017–October 2020.

To identify eligible studies in the electronic databases, two independent reviewers (SBDT/CJC) screened the titles and abstracts. They assessed the full text of selected citations in detail against the inclusion criteria and recorded the reasons for exclusion. Disagreements that arose between the reviewers were resolved through discussion with a third reviewer (CM). Additional eligible studies were searched for in the references of retrieved articles. The results of the search and the study inclusion process were reported in a Prisma flowchart [14].

#### Extraction of Data and Presentation of Findings

The reviewers developed a data extraction tool to extract data of included SRs including goal, context, study methods, methodological quality appraisal, and key findings relevant to the review question. They were summarized in a table and described narratively.

To categorize the measures used to reduce sugar consumption, we employed categories based on those of Kirkpatrick et al. (“interventions influencing price,” “interventions influencing changes to the food environment,” and “health promotion and education interventions”), and the NOURISHING framework. This latter formalizes possible policies to promote a healthy diet across three domains (food environment, food system, and behavior change communication), and 10 sub-policies areas represented by the letters of the word NOURISHING [10], shown in Supplementary File S3.

We defined the following categories: 1) economic tools including taxes, 2) product reformulation and labels, and 3) education/healthy food environment [12]. The first category corresponds to the “intervention influencing price” of Kirkpatrick et al. and to the letter U of NOURISHING. For the second category, we have combined the different measures from the food environment domain of NOURISHING (letters I and N) other than economic tools, and that we consider applicable at a macro-level (regional or national level). No findings were available for letters R and S. Our last category combines the measures that aim to offer healthy food in specific settings (letter O of “the food environment” domain) and information and education measures (letters I and G of the “behavior change communication” domain). This combination, from two different domains, may seem counter-intuitive as these interventions are expected to impact sugar intake via different pathways. However, as widely recommended, these two types of measures were combined in the large majority of SRs that we included in our scoping review. When feasible, the findings of the SRs were further separated depending on the type of measure.

### Search of Measures Implemented in European Countries in the NOURISHING Database

We searched all measures implemented in European countries that aimed to decrease sugars intake among populations in the NOURISHING database (last search conducted in October 2020) [11]. We focused on Europe in order to provide a clear overview of the measures implemented in this world region instead of providing unmanageable data for all countries across the world. We synthesized the results in a table using the NOURISHING framework [10].

## Results

### Characteristics of Included Studies

**FIGURE 1:**
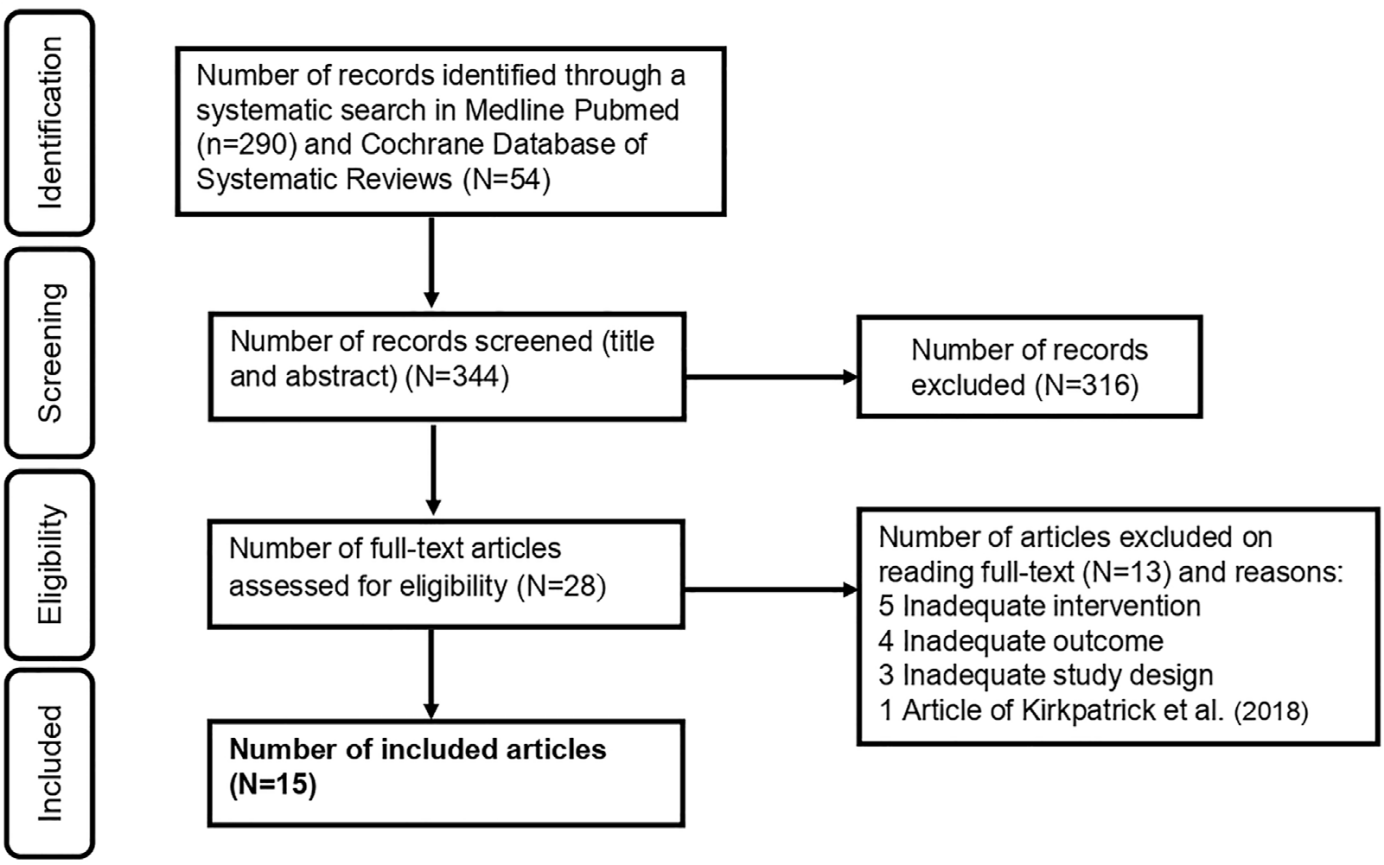
Prisma figure showing the inclusion and exclusion of systematic reviews (Impact of measures aiming to reduce sugars intake in the general population and their implementation in Europe: a scoping review. Switzerland. 2019–2021).

After screening 292 titles and abstracts, and reading 53 full texts, we have included a total of 15 SRs in the current review, as described in Figure 1.

The 15 SRs included in this scoping review assessed the effect of the following interventions on sugar intake:

1) Economical tools: five SRs addressed this question i.e., two SRs focusing only on SSB [15], one only on foods with added-sugar [16] and two included both [17]. These SRs included studies with different designs, such as laboratory studies, modelling studies, comparison studies, and pre-post implementation.
2) Product reformulation and labels: three SRs assessed the impact of product reformulation [6, 18, 19], and three SRs assessed the impact of labels or claims [6, 20, 21]. Heterogeneous outcomes were studied such as knowledge, purchase intention, purchase/sale, consumption, diet quality, sugar or energy intake and body weight.
3) Education/healthy food environment: Five SRs evaluated the effect of educational and healthy food environmental interventions [22–26] and two SRs studied the impact of healthy food environmental interventions only [6, 27]. In these seven SRs, the settings were varied and included school, home and community, and a clinical setting. The types of interventions included nutrition education, incentivizing healthier options, reducing availability of less healthy options, policy implementation, and providing water.

The authors of the included SRs assessed the quality of the primary studies using different tools, mainly the Cochrane risk-of-bias tools [28, 29]. Two studies did not provide information on quality assessment [24, 30]. Table 1 shows the characteristics of the 15 SRs included.

**TABLE 1:** Characteristics of the 13 included systematic reviews (Impact of measures aiming to reduce sugars intake in the general population and their implementation in Europe: a scoping review. Switzerland. 2019–2021).

Authors and year	Aim of the review	Period covered	Relevant (total) studies (n)	Interventions considered	Study designs considered	Quality appraisal and/or risk of bias	Funding
Characteristics of the included systematic reviews *on economic tools*							
[16]	To assess the effects of taxation of unprocessed sugar or sugar-added foods in the general population on the consumption of unprocessed sugar or sugar-added foods, the prevalence and incidence of overweight and obesity, and the prevalence and incidence of other diet related health outcomes	Up to October 2019	1 (1)	Taxes on or artificial increases of selling prices for unprocessed sugar or food products that contain added sugar	Controlled studies with more than one intervention or control site and interrupted time series studies with at least three data points before and after the intervention	The EPOC-adapted Cochrane “Risk of bias” tool	Scottish Institute for Research in Economics (SIRE) Early Career Engagement Grant
						GRADE approach	
[6]	To assess the effects of environmental interventions (excluding taxation) on the consumption of sugar-sweetened beverages and sugar-sweetened milk, diet-related anthropometric measures and health outcomes, and on any reported unintended consequences or adverse outcomes	From databases inception to January 24, 2018	7 (58)	Economic tools (price increases on SSB, financial incentives to purchase low-calorie beverages, price discounts on low-calorie beverages in community stores)	Comparison before and after economic tools implemented	The EPOC-adapted Cochrane “Risk of bias” tool	No specific grant
						GRADE approach	
[15]	To conduct a systematic review of real-word SSB tax evaluations and examine the overall impact on beverage purchases and dietary intakes by meta-analysis	From database inception to June 2018	18 (18)	SSB^a^ taxes	Comparison between pre–post tax (n = 11) or taxed and untaxed jurisdiction(s) (n = 6)	Critical appraisal tool based on 12 study quality criteria	Health Research Council (HRC) of New Zealand and the BODE3 Program
[17]	To examine research evidence on the health and behavioral impacts of fiscal measures targeted at high sugar foods and SSBs in both adult and children populations	2010 - October 2014	11 (11)	High sugar foods and SSBs^a^ taxes	Laboratory (n = 4), virtual setting (n = 4), controlled field experiments in supermarkets (n = 2) or a cafeteria (n = 1)	Joanna Briggs Institute appraisal tools	Public Health England (PHE)
[30]	To determine how health taxes can be designed to reduce consumption of targeted products and related health harms (and two other aims non-related to our question)	1990 - May 2016	49 (102)	Health taxes that target unhealthy products, including SSB^a^	Modelling studies (n = 54); experiments (n = 10), public opinion surveys (n = 9), qualitative approaches (n = 11) and mixed methods (n = 2)	Not stated	Cancer Research UK and Phillip Leverhulme Prize award
Characteristics of the included systematic reviews on *product reformulation and labels*							
[6]	To assess the effects of environmental interventions (excluding taxation) on the consumption of sugar-sweetened beverages and sugar-sweetened milk, diet-related anthropometric measures and health outcomes, and on any reported unintended consequences or adverse outcomes	From databases inception to January 24, 2018	11 (58)	Labelling and whole food supply	Comparison before and after labelling and whole food supply interventions implemented	The EPOC-adapted Cochrane ‘Risk of bias’ tool	No specific grant
						GRADE approach	
[20]	To summarize the evidence for the association between use of food labels and dietary intake	1995–2016	36 (36)	general food labels, nutrition facts panel, serving sizes, ingredients list, front-of-pack labels, health-related claims	20 cross-sectional, 1 cohort, 5 RCTs^b^	Academy of Nutrition and Dietetic Quality Criteria Checklist	No specific grant
[18]	To determine the effect of product reformulation measures on sugar intake and health outcomes	1990 to early 2016	16 (16)	Product reformulation	4 RCTs^b^, 6 modeling studies, 5 simulation studies, and 1 mix study	The Cochrane risk-of-bias tools	No specific grant
[21]	To assess the influence of nutrition claims relating to fat, sugar, and energy content on product packaging on several aspects of food choices to understand how they contribute to the prevention of overweight and obesity	January 2003 to April 2018	2 (11)	Nutrition claims related to sugar content (1 study on cereal and 1 study on yogurt)	Experimental setting	Effective Public Health Practice Project’s Quality Assessment Tool for Quantitative Studies	No specific grant for this research
[19]	To undertake a systematic review of simulation studies that model dietary strategies aiming to improve nutritional intake, body weight, and related chronic disease, and to assess the methodologic and reporting quality of these models	From database inception to July 2016	2 (45)	Product reformulation	Modeling studies	Methodology and reporting quality critiqued with a set of quality criteria adapted from generic modeling guidelines	National Health and Medical Research Council Program Grant (631947). Early Career Fellowship (1053359)
Characteristics of the included systematic reviews on *education/environmental interventions*							
[22]	To examine whether the promotion of water intake could reduce sugar sweetened beverage (SSB^a^) consumption or purchases independent of interventions that target SSBs^a^	January 2000 -January 2019	17 (17)	Water provision, education or promotion activities	9 RCTs^b^, 6 nonrandomized controlled trials, and 2 single-group pre-post studies	The Cochrane Collaborative Risk of Bias 2.0 and Risk Of Bias In Nonrandomized Studies-I tools	Healthy Eating Research, a national program of the Robert Wood Johnson Foundation
[6]	To assess the effects of environmental interventions (excluding taxation) on the consumption of sugar-sweetened beverages and sugar-sweetened milk, diet-related anthropometric measures and health outcomes, and on any reported unintended consequences or adverse outcomes	From databases inception to January 24, 2018	23 (58)	Nutrition standards in public institutions (schools) and home-based	Comparison before and after the implementation of nutrition standards in public institutions (schools) and home-based interventions	The EPOC-adapted Cochrane “Risk of bias” tool	No specific grant
*Interventions on environment only*						GRADE approach	
[23]	To explore the effectiveness of educational and behavioral interventions to reduce SSB^a^ intake and to influence health outcomes among children aged 4–16 years	From database inception to September 2016	16 (16)	12 school-based interventions and 4 community or home interventions	16 RCTs^b^: 12 school-based interventions and 4 community or home interventions	The Cochrane risk-of-bias tool	University of Balamand, Lebanon and World Health Organization, University of Liverpool, UK
[27]	To systematically review and quantify the impact of school food environment policies on dietary habits, adiposity, and metabolic risk in children	From database inception to December 2017	8 (8)	School food environment policies targeting food/beverage availability at school	1 RCT^b^ and 7 non-randomized trials	Assessment of exposure, outcome, control for confounding, and evidence of selection bias	NIH, NHLBI
*Interventions on environment only*							
[24]	This review aimed to scope the literature documenting SSB^a^ consumption and interventions to reduce SSB^a^ consumption among Australian Aboriginal and Torres Strait Islander people	From January 1980 to June 2018	18 (18)	Intervention that had a specific focus on reducing SSB^a^ consumption: incentivizing healthier options (n = 4), reducing availability of less healthy options (n = 1), nutrition education (n = 5), multifaceted (n = 5) or policy implementation (n = 3)	Interventional studies	No quality assessment	University of South Australia and the NHMRC Program
[25]	To evaluate the effectiveness of public health interventions to reduce SSB^a^ intake or increase water intake in children, adolescents and adults. To examine the study characteristics that could bring about change in consumption patterns	1990–2016	50 in total	Education, including in clinical setting (n = 27)	RCTs^b^	The Cochrane risk-of-bias tools	One author is supported by governmental scholarship
			40 in the meta-analysis	Education and delivery of water (n = 5)	cluster RCTs^b^		
				Education and environmental changes (n = 3)	non-RCT^b^ on community-based interventions with control group		
				Only delivery or environmental change or a mix (n = 3)			
[26]	To verify the efficacy of school-based interventions aimed at reducing SSB^a^ consumption among adolescents in order to develop or improve public health interventions	From database inception to December 2016	36 (36)	Educational/behavioral interventions (n = 20)	RCTs^b^ or cluster RCTs^b^ (n = 13)	Effective Public Health Practice Project (EPHPP) tool	University of Quebec, Canada
				Legislative/environmental interventions (n = 10)	Quasi-experimental studies (n = 11)		
				Intervention targeting both individuals and their environment (n = 6)	One-group pre-post studies (n = 12)		

a Sugar-sweetened beverages.

b Randomized controlled trial.

### Impact of the Use of Economic Tools Mainly Health Taxes

Table 2 details the findings of the five SRs that assessed the impact of economic tools on sugar consumption. Pfinder et al. showed that taxing foods exceeding a specific sugar threshold value impacted the consumption of sugar-added foods based on one study [16]: after implementation of the Hungarian public health product tax, the mean consumption of taxed sugar-added foods decreased by 4.0% [95% Confidence interval (CI): −0.07 to −0.01; very low-certainty evidence]. Teng et al., who made comparison between pre–post tax (n = 11) or taxed and untaxed jurisdiction(s) (n = 6), found that a 10% SSB tax was associated with an average decline in beverage purchases and dietary intake of 10.0% (95% CI: −5.0% to −14.7%), with considerable heterogeneity between results between jurisdictions [15]. Meta‐analysis results varied by study design and tax type, but not significantly. No significant difference was observed by study quality, consumption measure, age group (all ages, adults or children), or funding source.

**TABLE 2:** Overview of evidence on the effect of economic tools, product reformulation and labels, and educational/environmental interventions (Impact of measures aiming to reduce sugars intake in the general population and their implementation in Europe: a scoping review. Switzerland. 2019–2021).

Authors, year	N studies and location	Type of interventions	Study population	Mains conclusions regarding effectiveness	Key findings related to offsetting or compensatory behaviors	Main limitations
Evidence on the effect of *economic tools*						
[16]	1	Taxes on or artificial increases of selling prices for unprocessed sugar or food products that contain added sugar	Children (0–17 years) and adults (18 years or older)	There was very limited evidence and the certainty of the evidence was very low		The results of this systematic review was derived from one included study, resulting in low evidence
	Hungary			The mean consumption of taxed sugar-added foods (measured in units of kg) decreased by 4.0% (95% CI: −0.07 to −0.01)		
[6]	7	Economic tools (price increases on SSB^a^, financial incentives to purchase low-calorie beverages, price discounts on low-calorie beverages in community stores)	Children, teenagers and adults	Price increases on SSBs^a^ were associated with decreasing SSB sales, but with a moderate-certainty evidence. Based on three studies, SSB^a^ sales decreased by −19% (95% CI −33 to −6) at 4–12 months		Few studies have evaluated the same interventions, so the evidence is low
	Mostly United States, but also Australia, the Netherland, Canada, UK and others			Price discounts on low-calorie beverages resulted in mixed effects on SSB^a^ sales		
[15]	18	SSB^a^ taxes	Children and adults	Meta-analysis	A 10% SSB^a^ tax was associated with a nonsignificant 1.9% increase in total untaxed beverage consumption (e.g., water) (95% CI: −2.1 to 6.1%)	Risk that incomplete publications biased results towards a greater decline
	Mostly US and Europe			A 10% SSB^a^ tax was associated with an average decline in beverage purchases and dietary intake of 10.0% (95% CI: −5.0% to −14.7%), with considerable heterogeneity between results		
[17]	11	High sugar foods and SSBs^a^ taxes	Adults (n = 10) Children 12–14 years (n = 1)	In 10 studies, an increase in the price of high sugar foods and SSBs^a^ resulted in a decrease in purchases, at least in the short term. This reduction may be proportionate to the level of price increase imposed. One study showed no effect	In two studies, subsidies of “healthy” foods lead to an increase in “unhealthy” and/or total calories purchase	Eight studies were conducted in a laboratory or virtual setting which may not reflect real-life situations
	United States (n = 7), Europe (n = 4)					None of the studies examined the effects of pricing on consumption or longer term health outcomes
[30]	102, mostly US (n = 51) and Europe (n = 34)	SSB^a^ taxes	Children and adults	18/26 studies found a positive impact of SSBs^a^ taxes on reduction in their consumption		The authors could not apply a uniform method of critical appraisal across studies
				High tax on SSBs^a^ (>20% of the initial price) are more likely to have a positive impact on health behaviors and outcomes		
Evidence on the effect of *product reformulation and labels*						
[6]	11	Labelling and whole food supply	Children, teenagers and adults	Moderate-certainty evidence: traffic-light labelling was associated with decreasing SSB^a^ sales		Few studies have evaluated the same interventions, so the evidence is low
*Product reformulation and label/claims*	Mostly United States, but also Australia, Netherlands, Canada, UK and others			Low-certainty evidence: nutritional rating score labelling was associated with decreasing sales of SSBs^a^		
				For menu-board calorie labelling reported effects on SSB^a^ sales varied		
				Associations between voluntary industry initiatives to improve the whole food supply and SSB^a^ sales varied		
[20]	36	General food labels, nutrition facts panel, serving sizes, ingredients list, front-of-pack labels, health-related claims	Adults	Food labels: 12/13 studies found positive association with healthier diet quality. One study found a negative association	Health related claims may lead to overconsumption, known as the “health halo” effect	Most studies had neutral ratings on quality checklist
*Label/claim*	Mostly United States (n = 20), but also Europe (n = 4), South Korea (N = 2) and Australia (n = 1)			Nutrition facts panels: 10/12 studies found positive association with a healthier diet.		Limitations of the included studies: non-generalizable demographics, use of observational studies, risk of self-selection bias and social desirability bias
				Ingredients lists, serving size information and front-of-pack labels: insufficient research		
				Health related claims: unclear whether beneficial or detrimental		
[18]	US (n = 7), Canada (n = 1), UK (n = 3) and France (n = 3)	4 RCTs^b^, 6 modeling studies, 5 simulation studies, and 1 mix study	Children, teenagers and adults	Results from RCTs^b^ showed that consumption of reformulated products can reduce sugar intake and body weight		Limited number of included studies with high risk of bias and an overall low to very low quality grade
*Product reformulation*				Meta-analysis:		Limitations of the included studies: inadequacy of dietary intake and food composition data, variation in the interventions
				Reformulation of products lead to a reduction of −11% (95% CI, −20 to 2) in sugar intake and −1.0 kg (95% CI, −2.2 to −0.1) in body weight		
[21]	2	Nutrition claims related to sugar content (1 study on cereal and 1 study on yogurt)	Adults	Findings indicated that nutrition claims may have an impact on the knowledge of consumers with respect to perceived healthful- ness, expected and experienced tastiness, and perceived appropriate portion size. Nutrition claims were found to potentially influence food purchase intentions, food purchases and consumption	The findings also indicated the potential for unintended consequences, whereby nutrition claims may lead to overconsumption of foods and subsequent higher energy intakes	Low methodological quality of the included studies
*Claims*	Germany (n = 2)					Results may vary depending of the type of food tested (healthier or unhealthy). Only a few studies measured energy intake
[19]	9	Simulation studies on reformulation (n = 9): e.g., reformulation of foods to reduce nutrient content	Children and adults (2–69 years old)	Reformulating SSBs^a^ by replacing added sugar with artificial sweetener theoretically reduced energy intake by 107 kcal/d. Reformulating free sugars in SSBs^a^ without the addition of artificial sweeteners at a level of 9.7% per year predicted a 38 kcal/d reduction in energy intake over 5 years		Lack of quality assessment tools specifically designed for dietary simulation modeling studies
*Product reformulation*	Netherlands (n = 3), UK, Finland, US (n = 2), Australia, New Zealand					Limited external validity due to the design of simulation studies
Evidence on the effect of *educational/environmental interventions*						
[22]	17	Water provision, education, and promotion activities. 11/17 studies used 2 or more types of interventions	Children ages 2–18 years in 14 studies and adults in three studies	In 7/17 studies, a statistically significant decrease in SSB^a^ consumption/purchase was observed		Only two included studies were at low or some/moderate risk of bias
	Europe (n = 8), United States (n = 6), Australia (n = 2), and in the Caribbean (n = 1)	Intervention settings included schools (9), homes (3), supermarkets (2), other child-focused settings such as preschools (2), and community-wide (2)		Studies that included water provision, education or promotion, or some combination reported decreased SSB^a^ intake more often than water price discounting and community intervention studies, which had no effects		A large heterogeneity was observed in the measurement of SSB^a^ intake
[6]	11	Nutrition standards in public institutions (schools) and home-based	Children, teenagers and adults	Low-certainty evidence: reduced availability of SSBs^a^ in schools was associated with decreased SSB consumption		Few studies have evaluated the same interventions, so the evidence is moderate-low
*Interventions on environment only*	Mostly United States, but also Australia, the Netherland, Canada, UK and others			Low-certainty evidence: improved availability of drinking water in schools and school fruit programs were associated with decreased SSB^a^ consumption. Reported associations between improved availability of drinking water in schools and student body weight varied		
				Improved availability of low-calorie beverages in the home environment was associated with decreased SSB^a^ intake (by –413 ml/day, 95% CI –684 to –143 at 4–12 months, moderate-certainty evidence) and with decreased body weight among adolescents with overweight or obesity and a high baseline consumption of SSBs^a^ (high-certainty evidence)		
[24]	18	Intervention aiming to reduce SSB^a^ intake: incentivizing healthier options (n = 4), reducing availability of less healthy options (n = 1), nutrition education (n = 5), multifaceted (n = 5) or policy implementation (n = 3)	Australian Aboriginal and Torres Strait Islander people	The more impactful studies seemed to be those which were community driven or involved extensive community consultation and collaboration		An appraisal of quality of included study was not performed
	Remote communities (n = 13), rural communities (n = 1), South East Queensland (n = 2) and Victoria (n = 2) in Australia			Findings from the effect of educational-interventional programs were controversial		
[27]	North America: US (n = 7), Canada (n = 1)	Competitive food/beverage standard provided at school: product-specific restrictions; standards on nutrients, calories, or portion sizes; or both	Children in primary, secondary and preschools	Meta-analysis: Competitive food/beverage standards reduced habitual intake SSBs^a^ by 0.18 servings/day (95% CI: −0.31, −0.05) and unhealthy snacks by 0.17 servings/day. SSB in-school intake decreased by −0.02 servings/d (n = 5), but it was not significant (95% CI: −0.04, 0.01)	No effect on total calories was observed	Some studies were judged to have lower quality scores
*Interventions on environment only*						Several studies included other intervention components that might contribute to impact
[23]	16	12 school-based interventions and 4 community or home interventions	Children aged 4–16 years	Overall, educational and behavioral interventions, when compared with no intervention, were found to be successful in reducing SSB^a^ intake among children and adolescents		13/16 eligible trials (= 17′555 participants) could not be included in the meta-analysis because of the variability in scales used to report the outcomes of interest
	Mostly Europe (n = 10) and US (n = 4)			Meta-analysis (n = 3):		
				School-based interventions were associated with a trend toward reduction in SSB^a^ intake compared with no intervention (−284 ml; 95% CI, −643 to 76)		
				Body mass index z scores did not change significantly		
[25]	50 studies from United States, Europe, South America, Australia, Canada, Malaysia, New Zealand	Education, including in clinical setting (n = 27)	Children, adolescents and adults	Meta-analysis (n = 40):	Among behavior change techniques used, the technique “model/demonstrate the behavior” was associated with the greater effectiveness to reduce SSB^a^ across all age groups	Risk of bias across the 40 studies meta-analyzed was generally medium to high
		Education and delivery of water (n = 5)		Interventions significantly decreased consumption of SSB^a^ in children by 76 ml per day [95% confidence interval (CI) −105 to −46; 23 studies, *p* < 0.01], and in adolescents (−66 ml per day, 95% CI −130 to −2; 5 studies, *p* = 0.04) but not in adults (−13 ml per day, 95% CI −44 to 18; 12 studies, *p* = 0.16)		
		Education and environmental changes (n = 3)		For children, there was evidence to suggest that modelling/demonstrating the behavior helped to reduce SSB^a^ intake and that interventions within the home environment had greater effects than school-based interventions		
		Only delivery or environmental change or a mix (n = 3)				
[26]	United States and Canada (n = 27), Europe (n = 3), Australia (n = 2), Brazil (n = 1), Asia (n = 3)	Educational/behavioral interventions (n = 20)	Adolescents (12–17 years old)	Over 70% of all interventions, targeting individuals, their environment or both, were effective in decreasing SSB^a^ consumption. The success rates were 90% for legislative/environmental approaches; 65% for educational/behavioral interventions; and 67% for a combination of educational/behavioral and legislative/environmental approaches	Two studies reported significant increases in SSB^a^ consumption post-intervention	Large heterogeneity between studies, preventing from conducting a meta-analysis
		Legislative/environmental interventions (n = 10)				
		Intervention targeting both individuals and their environment (n = 6)				

a Sugar-sweetened beverages.

b Randomized controlled trial.

Von Philipsborn et al. demonstrated, based on three studies, that an increased SSB price was associated with a reduction in SSBs sales by −19% (95% CI: −33 to −6%) at 4–12 months [6]. Roberts et al. found in 10/11 studies a decrease in purchases of SSBs and high sugar foods, at least in the short term, following an increase in prices [17]. Wright et al. observed in 18/26 studies a positive impact of SSB taxes on the reduction of their consumption. The observed effect may be proportionate to the level of the tax. Tax on SSBs higher than 20% of the initial price appeared more likely to have a positive impact [30].

### Impact of the Use of Product Reformulation or Labels/Claims

The two SRs of Hashem et al. and Grieger et al. assessing the impact of reformulation essentially included modelling and simulation studies (Table 2) [18, 19]. They showed a theoretical reduction in sugar consumption and an estimated improvement in health outcomes. In addition, Hashem et al. observed, based on four randomized controlled trials assessing the effect of sugar-reformulated products over a period of 8–10 weeks, a reduction of −11% (95% CI, −20 to 2) in sugar intake and −1.0 kg (95% CI, −2.2 to −0.08) in body weight [18]. In the SR of von Philipsborn et al., very low-certainty evidence from three studies suggested that voluntary industry initiatives to improve the nutritional quality of the whole food supply may affect SSB sales and purchases, but the direction of reported effects varied [6].

Regarding the use of food labels, the SR of von Philipsborn et al. found moderate-certainty evidence that traffic-light labelling was associated with decreasing sales of SSBs, and low-certainty evidence that nutritional rating score labelling was associated with decreasing sales of SSBs [6]. Anastasiou et al. found a positive association between food labels and healthier diet quality in 12/13 studies, and one study found a negative association. In addition, the authors observed a positive association between the use of nutrition fact panels and a healthier diet in 10/12 studies. Research on the effect of the use of ingredient lists, serving size information and front-of-pack labels was insufficient to draw conclusions [20].

In the two SRs of Oostenbach et al. and Anastasiou et al., it remains unclear whether the impact of food health-related claims was beneficial or detrimental [20, 21].

### Impact of the Educational and Healthy Food Environmental Interventions

As stated previously, the seven SRs assessing the impact of educational/healthy food environmental interventions included very heterogenous study designs, interventions, settings, and populations. Except the SR of Micha et al. focusing on SSB and snacks, the other SRs evaluated only SSB intake, as illustrated in Table 2.

The majority of those reviews observed a reduction in sugar intake, mostly assessed by a reduction in SSB consumption. The SRs of Micha et al. [27] and von Philipsborn al et. [6], which studied the impact of healthy food environmental interventions only, and not education, showed beneficial impact on SSB intake, and unhealthy snacks intake or weight. More specifically, in a meta-analysis including 5/11 studies, von Philipsborn et al. reported a decrease in SSB consumption of −413 ml/day (95% CI: −684 to −143) after 4–12 months, when improving access to low-calorie beverages in the home environment among high consumers of SSBs at baseline. The same SR also concluded that a reduced availability of SSBs in schools was associated with decreased SSB consumption. Based on low evidence, these authors showed that improved availability of drinking water in schools and school fruit programs were associated with decreased SSB consumption [6]. Micha et al. reviewed the impact of school food environmental policies and observed a reduction of 0.18 servings of SSB/day (95% CI: −0.31 to −0.05) compared to habitual intake [27].

The five other included SRs examined the effect of educational and healthy food environmental interventions. In 2020, Dibay Moghadam et al. found a statistically significant decrease in SSB consumption/purchase in 7/17 (41%) studies promoting water consumption [22]. Based on 36 studies, Vézina-Im et al. concluded that over 70% of all interventions targeting individuals, their environment, or both were effective in decreasing SSB intake. The success rates (defined as the proportion of studies showing a significant reduction in SSB consumption) were 90% for legislative/environmental approaches, 65% for educational/behavioral interventions, and 67% for a combination of educational/behavioral and legislative/environmental approaches [26]. These authors found that more than half of the interventions were based on a psychosocial theory. The most frequent behavior-change techniques were: providing information about health consequences, restructuring the physical environment, behavioral goal setting, self-monitoring of behavior, threats to health, and providing general social support [26].

Some SRs provided findings for specific age groups. Abdel Rahman et al. assessed the impact of educational and behavioral interventions among children aged 4–16 years and found a trend toward reduction, with a mean reduction of 284 ml/day (95% CI, −643 to 76) [23]. Vargas-Garcia et al. evaluated the impact of educational and/or environmental changes on different age groups. A significant decrease in SSB intake was observed in children, with a mean reduction of 76 ml/day (95% CI: −105 to −46; 23 studies), and in adolescents, with a mean reduction of 66 ml/day (95% CI: −130 to −2; 5 studies). The reduction in adults was not statistically significant [25].

### Comparison With the Previous Findings

A total of 12 SRs were included in the review of Kirkpatrick et al. [12], assessing the effect of price changes (n = 6) [31–36] and food environment interventions (n = 7) [34, 37–42], and health promotion and education (n = 7). The seven SRs that assessed the effect of food environmental interventions were the same that studied the impact of health promotion/education. The Supplementary File S4 details the characteristics and main findings of the studies included in the review of Kirkpatrick et al., as presented by these authors.

As illustrated in Table 3, Kirkpatrick et al. found positive impact of taxes on SSB demand and consumption in four SRs [12]. The findings on taxes and weight changes were less consistent; two reviews found positive outcomes, two found mixed results, and one showed no effect. No SR on the impact of labels/reformulation were included by Kirkpatrick el al. [12]. The five SRs that assessed the impact of environmental interventions alone showed reduction in SSB consumption. The impact of educational measures on SSB consumption was positive in three SRs, mixed in one SR, and no impact was observed in one SR. The impact of the educational/environmental measures on weight was more contrasted (beneficial effects: n = 3; mixed effects: n = 4; and no effect: n = 2).

**TABLE 3:** The different types of measures studied in the systematic reviews and their impact on sugar-sweetened beverages demand, sugar-sweetened beverages consumption, weight outcomes, and other outcomes. (Impact of measures aiming to reduce sugars intake in the general population and their implementation in Europe: a scoping review. Switzerland. 2019–2021).

	SSB^a^ demand	SSB^a^ consumption	Weight outcomes	Other outcomes
Economic tools				
[16]				+ (sugar-added foods intake)
[6]	+			
[15]	+			
[17]	+			
[30]		+	+	
*[31]			+	
*[32]			+/Ø	
*[33]	+		+	
*[34]		+	−	
*[35]		+		
*[36]	+	+	+/Ø	
Label/reformulation				
[6]	+ labelling			
	+/- reformulation			
[20]				+/− (healthier diet quality)
[18]			+	+ (sugar intake)
[21]	+/Ø			
[19]				+ (energy intake)
Environment only				
[6]		+	+	
[27]		+		+ (unhealthy snack intake)
*[38]		+	+/Ø	
*[37]		+		
*[40]		Ø	+/Ø	
*[39]		+	+/Ø	
*[34]		+/Ø		
*[41]		+	+	
Education only				
*[38]		+	+/Ø	
*[37]		+		
*[40]		Ø	+/Ø	
*[39]		+	+/Ø	
*[34]		+/Ø		
Education/Environment				
[22]		+/Ø		
[24]		+/−		
[23]		+	Ø	
[25]		+ children and adolescents		
		Ø adults		
[26]		+/−		
*[42]		+/Ø		

a Sugar-sweetened beverages.

Authors with an asterisk are the SRs included in the review of SRs of Kirkpatrick et al. [12].

+ Beneficial effects; reduction in SSB consumption or weight outcomes observed in the large majority of studies.

Ø No effect: no reduction in SSB consumption or weight outcomes observed.

− Negative effect: increase in SSB consumption or weight outcomes observed.

+/Ø or +/−: both beneficial and no effect/negative effect observed.

**FIGURE 2:**
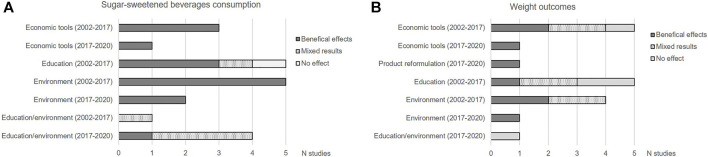
Impact on the different types of measures on sugar-sweetened beverages consumption **(A)** and weight outcomes **(B)** in the systematic reviews included in the review of Kirkpatrick et al. and in the reviews published between 2017 and 2020. (Impact of measures aiming to reduce sugars intake in the general population and their implementation in Europe: a scoping review. Switzerland. 2019–2021).

As shown in Figure 2, considering all types of measures, a beneficial impact on SSB consumption was observed in 15/21 SRs included in the review of Kirkpatrick et al. and published since 2017 [12]. Beneficial effects on SSB consumption were observed for economic tools and interventions focusing on environment alone whereas SRs on education and those combining educational and environmental interventions found mixed results. Regarding the impact on weight outcomes, the results were less concordant. In the most recent SRs, economic tools, reformulation, and environmental interventions showed beneficial effects.

### Synthesis of NOURISHING Database

Several European countries have implemented measures aimed at reducing sugar consumption in the population. In the “Behaviour change communication” domain, information (I) and nutritional education (G) are the most widely implemented measures, as described in Table 4. In the “Food environment” domain, the most frequently implemented measures are labels (N), followed by environmental measures aiming to offer healthy food and set standards in public institutions and other specific settings (O), restricting food advertising and other forms of commercial promotion (R), and health-related food taxes (U). In contrast, only a few countries have implemented environmental measures in the following areas: improving the nutritional quality of the whole food supply, including reformulation (I) and setting incentives and rules to create a healthy retail and food service environment (S). In the “Food system” domain, only a few countries have implemented measures aiming at decreasing sugar intake in the population.

**TABLE 4:** The different types of measures to decrease sugar intake implemented in countries according to NOURISHING (Impact of measures aiming to reduce sugars intake in the general population and their implementation in Europe: a scoping review. Switzerland. 2019–2021).

Nourishing classification	Implemented measures	Countries where implemented
N	Nutrition label standards and regulations on the use of claims and implied claims on food	Croatia, Denmark, EU countries, France, Iceland, Liechtenstein, Lithuania, Norway, Sweden, Switzerland, United Kingdom
O	Offer healthy food and set standards in public institutions and other specific settings	Austria, Belgium, Bulgaria, Estonia, Finland, France, Germany, Hungary, Latvia, Lithuania, Malta, Norway, Poland, Portugal, Romania, Slovenia, Spain, Sweden, United Kingdom
	• Mandatory standards for food available in schools including restrictions on unhealthy food	
	• Mandatory regulation of food advertising on non-broadcast communications channels	
	• Mandatory regulation of food advertising through any medium	
	• Voluntary guidelines for food available in schools	
	• Bans specific to vending machines in schools	
	• Standards in other specific locations (e.g., health facilities workplaces)	
U	Use economic tools to address food affordability and purchase incentives	Belgium, Estonia, Finland, France, Hungary, Ireland, Latvia, Norway, Portugal, Spain, United Kingdom
	• Health-related food taxes	
	• Voluntary health-related foods taxes	
R	Restrict food advertising and other forms of commercial promotion	Belgium, Denmark, European Commission, Finland, Hungary, Ireland, Latvia, Norway, Portugal, Spain, Sweden, Switzerland, United Kingdom
	• Mandatory regulation of broadcast food advertising to children	
	• Voluntary regulation of food advertising on non-broadcast communications channels	
	• Governmental engage with industry to develop self-regulation to restrict food marketing to children	
	• Government support voluntary pledges developed by industry	
I (Improve)	Improve nutritional quality of the whole food supply	France, Norway, Switzerland, United Kingdom
	• Voluntary reformulation of food products	
S	Set incentives and rules to create a healthy retail and food service environment	United Kingdom, France, Norway
	• Initiatives to increase the availability of healthier food in stores and food service outlets	
	• Incentives and rules to offer healthy food options as a default in food service outlets	
	• Incentives and rules to restrict sugar-sweetened beverage consumption	
H	Harness food supply chain & actions across sectors to ensure coherence with health	Finland United, Kingdom
I (Inform)	Inform people about food and nutrition through public awareness	Done in most countries
	• Development and communication of food-based dietary guidelines	
	• Development and communication of guidelines for specific food groups	
	• Public awareness, mass media and informational campaigns and social marketing on healthy eating	
	• Public awareness campaigns specific to fruit and vegetables	
	• Public awareness campaigns concerning specific unhealthy food and beverages	
N	No specific measures aiming at decreasing sugar intake in the population	
G	Give nutrition education and skills	Done in most countries
	• Nutrition education on curricula	
	• Community-based nutrition education	
	• Cooking skills	
	• Initiatives to train school children on growing food	
	• Workplace or community health schemes	
	• Training for caterers and food service providers	

## Discussion

The aim of this scoping review was to identify the types of measures implemented and evaluated to decrease sugars intake in the population and to assess their impact. We included 15 SRs published since 2017 and that assessed economic tools (n = 5), product reformulation and labels (n = 5), environmental interventions (n = 2), and educational/environmental interventions (n = 5). Despite high heterogeneity in the 15 SRs included, we observed a beneficial impact of economic tools and interventions focusing on environment alone on sugar intake, mostly with respect to SSB consumption whereas SRs combining educational and environmental interventions found mixed results. The impact on weight outcomes was less frequently studied but still showed a beneficial trend, especially in the most recent SRs evaluating economic tools, product reformulation, and environmental interventions. According to the NOURISHING database, the most frequently implemented measures in Europe were information through public awareness, nutritional education, and labels.

Among the three types of measures that have been studied in the SRs, economic tools, focusing mostly on health taxes, were studied by five SRs. The implementation of taxes has shown to be effective in reducing SSB purchases and consumption. The SR of Roberts et al. has shown that the reduction in SSB purchases may be proportionate to the level of price increase imposed [17]. Wright el al. concluded that a tax on SSBs higher than 20% of the initial price was more likely to have a positive impact on health behaviors and outcomes [30]. According to the NOURISHING database, economic tools have been implemented in several European countries, including Belgium, Estonia, Finland, France, Hungary, Ireland, Latvia, Norway, Portugal, Spain, and the United Kingdom. The level of the tax and the ways it has been implemented (e.g., definition of the foods and drinks subject to the tax) differs widely between countries. The majority of available evidence has shown a beneficial impact on short-term outcomes, mainly on SSB sales/consumption; however, other positive and long-term impacts should not be underestimated. The implementation of taxes on sugar may provide an incentive for the food industry to develop and promote healthier products. Some studies have extrapolated the long-term effects of a tax on SSB and concluded that they could reduce rates of illness, including obesity [31], mortality rates, health costs and increase quality of life [43–45].

The second type of measures that have been studied in the included SRs are product reformulation and labels/claims. Only three SRs have recently assessed the impact of product reformulation [6, 18, 19], mainly based on simulation and model studies. The results were promising on estimated sugar reduction and health outcomes; however, these results need to be confirmed in the community. This type of measure has the advantage of reducing consumers’ intake of certain nutrients without requiring a conscious effort on their part. The impact of labels and claims is more contrasted. These mixed results may be explained by the large variability in existing labels and their confusing effect on consumers, who may be lost at the time of purchase [46, 47]. The effects of claims were unsure because of potential compensatory behaviors. The NOURISHING database shows that product reformulation has been implemented only in four European countries, i.e., France, Norway, Switzerland, and the United Kingdom. This type of measure may be difficult to implement, as it requires the involvement of several partners, mainly the food industry, and may present challenges in terms of foods technology. In contrast, the majority of European countries have already implemented labels and claims, which are often applied in the form of the nutritional composition available on food and drinks packages.

The third type of measures that have been studied in the included SRs are educational and environmental interventions, mostly implemented in a school setting. High heterogeneity was observed in the interventions that included educational initiatives, especially nutrition education curriculums, and/or environmental measures, such as the provision of healthful foods or beverages and quality standards for competitive foods and beverages. It is therefore difficult to assess the impact of each of these individual interventions, as they are frequently associated as recommended by current guidelines on prevention of overweight and obesity and health promotion. Educational measures place a lot of responsibility on individuals to make the final choice about their diet while the environmental conditions strongly influence food choices and adequate conditions clearly promote healthy choices. In this scoping review, the findings of environmental interventions alone were more striking than education alone or a combination of educational/environmental measures. Educational interventions still remain important tools in order to improve the nutritional knowledge and food literacy of the population, and facilitate the acceptability of environmental measures. Therefore, both types of measures should be combined in a coherent approach in order to avoid nutritional aberrations resulting from the different messages and stakeholders. Monitoring of the measures is also crucial in order to validate and/or adapt these measures according to the results. Targeted evaluations of the measures in terms of the resources invested, the process, and the results obtained are essential. The NOURISHING framework may be useful for the various stakeholders to clarify the types of measures implemented and their coherence, and to structure the intervention in a comprehensive approach.

Our scoping review has some limitations. First, the SRs included in our review included mainly adults and children in a school setting, and some subgroups of the population were not represented, such as preschool children, pregnant women, and the elderly. Moreover, the studies were mostly conducted in the US and Europe, thus affecting the external validity of our findings. Secondly, we only searched for the measures implemented in European countries in the NOURISHING database and not those implemented worldwide. Thirdly, the quality of the original studies included in the SRs was often considered to have high risk of bias by the authors, which may affect the internal validity of the findings. The weaknesses of the studies were often related to data collection related to sugar intake, the definition of sugars, or the duration of interventions. In relation to the SRs included in our review, the study designs of included original studies were heterogenous, the measures to decrease sugar intake were also highly variable, and the outcome most frequently studied was reduction in SSB purchases and consumption. However, this intermediate outcome, widely used as a surrogate for total sugar intake, does not consider substitutions or compensatory behaviors.

In conclusion, this scoping review shows that three types of measures have been studied in SRs, including economic tools, product reformulation and labels, and education/environmental interventions. A high level of heterogeneity was observed in the methodologies, populations, and interventions in the original studies. Economic tools and environmental interventions were effective to reduce sugar intake, as mostly assessed by SSB purchases and consumption. Interventions combining educational and environmental measures found mixed results. The findings on weight outcomes were less concordant but still showed a positive trend, especially for product reformulation. Some of these measures are used in Europe, but the most frequently implemented measures to date are information through public awareness, nutritional education, and labels. To close the large gap between sugar intake recommendations and actual intake, future measures should be implemented based on the available evidence using a global approach and integrating a thorough long-term evaluation.
