# Supplementary material for: Impact of Measures Aiming to Reduce Sugars Intake in the General Population and Their Implementation in Europe: A Scoping Review
Source: Int J Public Health. 2022 Jan 13;66:1604108. doi: 10.3389/ijph.2021.1604108 (PMC8791851; doi:10.3389/ijph.2021.1604108)
Supplement: Supplementary file 1 [file DataSheet2.PDF]

Supplementary file 2

Search strategy on Medline PubMed

((((((review[Title]) OR (reviews[Title]) OR (synthesis[Title]) OR (overview[Title]) OR (meta analysis[Title]))) OR ((review[MeSH Terms]) OR (meta analysis[MeSH Terms])))) AND (((((prevent*[Title]) OR (program*[Title]) OR (strategy[Title]) OR (strategies[Title]))) OR ((intervention*[Title/Abstract]) OR (policy[Title/Abstract]) OR (policies[Title/Abstract]) OR (initiative*[Title/Abstract]) OR (guideline*[Title/Abstract]) OR (nutrition label*[Title/Abstract]) OR (calorie label*[Title/Abstract]) OR (reformulation*[Title/Abstract]) OR (nutrition* composition[Title/Abstract]) OR (nutrient list*[Title/Abstract]) OR (nutrition claim*[Title/Abstract]) OR (health claim*[Title/Abstract]) OR (nutrient content*[Title/Abstract]) OR (nutrient information[Title/Abstract]) OR (food label*[Title/Abstract]) OR (tax[Title/Abstract]) OR (taxes[Title/Abstract]) OR (taxation[Title/Abstract]) OR (fiscal*[Title/Abstract]) OR (price[Title/Abstract]) OR (pricing[Title/Abstract]) OR (prices[Title/Abstract]) OR (tariff*[Title/Abstract]) OR (subsid*[Title/Abstract]) OR (incentiv*[Title/Abstract]) OR (econometric*[Title/Abstract]) OR (advertis*[Title/Abstract]) OR (product placement[Title/Abstract]) OR (television[Title/Abstract]) OR (marketing[Title/Abstract]) OR (mass media[Title/Abstract]) OR (social media[Title/Abstract]) OR (campaign*[Title/Abstract]) OR (behaviour change[Title/Abstract]) OR (behavior change[Title/Abstract]))) OR ((child health services[MeSH Terms]) OR (health promotion[MeSH Terms]) OR (health education[MeSH Terms]) OR (primary prevention[MeSH Terms]) OR (preventive health services[MeSH Terms]) OR (child health services[MeSH Terms]) OR (health promotion[MeSH Terms]) OR (health education[MeSH Terms]) OR (primary prevention[MeSH Terms]) OR (preventive health services[MeSH Terms]) OR (school health services consumer health information health education, dental[MeSH Terms]) OR (health fairs[MeSH Terms]) OR (nutrition assessment[MeSH Terms]) OR (nutrition surveys[MeSH Terms]) OR (policy making[MeSH Terms]) OR (health policy[MeSH Terms]) OR (nutrition policy[MeSH Terms]) OR (food labeling[MeSH Terms]) OR (water*) OR (nutritive value[MeSH Terms]) OR (food/economics

[MeSH Terms]) OR (diet/economics[MeSH Terms]) OR (taxes[MeSH Terms]) OR (financing, government[MeSH Terms]) OR (marketing[MeSH Terms]) OR (mass media[MeSH Terms])))) AND ((((sweet*) OR (sugar*) OR (sucrose) OR (discretionary calorie*) OR (energy dense) OR (junk food) OR (soft drink*) OR (cake*) OR (pastry) OR (pastries) OR (biscuit*) OR (pudding*) OR (jam) OR (jams) OR (marmalade*) OR (confectioner*) OR (chocolate*) OR (energy drink*) OR (sports drink*) OR (sport drink*) OR (yogurt) OR (yoghurt) OR (breakfast cereal*) OR (juice*) OR (snack*) OR (candy) OR (candies) OR (dessert*) OR (fizzy) OR (soda) OR (pop))) OR ((Sucrose[MeSH Terms]) OR (sweetening agents[MeSH Terms]) OR (high fructose corn syrup[MeSH Terms])))

Limits:

Publications: systematic review, meta-analysis

Date: 01 June 2017 to October 2020
