# Supplementary material for: Impact of Measures Aiming to Reduce Sugars Intake in the General Population and Their Implementation in Europe: A Scoping Review
Source: Int J Public Health. 2022 Jan 13;66:1604108. doi: 10.3389/ijph.2021.1604108 (PMC8791851; doi:10.3389/ijph.2021.1604108)
Supplement: Supplementary file 2 [file DataSheet4.pdf]

Supplementary file 4

Table 1 : Caracteristics of the 12 systematic reviews included in the review of Kirkpatrick et al.

**Systematic reviews on the impact of taxes**					
**Authors and year**	**Aim of the review**	**Included studies (Total)**	**Population**	**Intervention**	**Main conclusion**
Backholer, 2016	1. To systematically review the literature for studies (of any study design) conducted in high-income countries that examined the effect of an SSB price increase on beverage purchase or consumption and/or weight outcomes according to an indicator of socioeconomic position.<br>2. We additionally aimed to examine the average amount paid in SSB tax across socio-economic strata (as a percentage of income).	11 (11)	Children and adults	1. Those that examined the association between variation in SSB taxes across US states and individual SSB consumption and/or BMI (n=3)<br>2. Price elasticity estimation of SSB demand from household food and beverage price and expenditure data (n=1)<br>3. Modelling to simulate the impact of a hypothetical SSB tax by combining price elasticity estimates with population data on the SEP specific patterning of beverage consumption, net energy intake or body weight outcomes (n=7)	SSB taxation is associated with improvements in population weight outcomes across socio-economic position groups or of a greater magnitude for lower compared with higher socio-economic position households
Bes-Rastrollo, 2016	We conducted a comprehensive literature review to include those systematic reviews based on the topic of sugar-sweetened beverages as a potential risk factor for weight gain or obesity.	24 (71)	Children and adults	Price changes and taxes	Studies based on simulations suggest an inverse relationship between taxes and weight gain/obesity, though magnitude of effects was small (though potential for benefit at the population level)<br><br>Observational studies (cross-sectional and cohorts) found no association between SSB taxes and weight gain

Cabrera Escobar, 2013	This review evaluates the published evidence for SSB taxes or price increases, and their potential impact on consumption levels and effects on obesity, overweight and BMI. The possibility of switching to alternative drinks is also considered.	9 (9)	Children and adults, all ages	Price changes and taxes	Higher SSB prices associated with lower SSB demand<br><br>Studies reporting weight outcomes too heterogeneous to be pooled but those from USA suggest that higher prices associated with decrease in BMI
Levy, 2011	1. This paper reviews the literature on both these sets of school policies directed at SSB consumption.<br>2. we also consider how SSB consumption affects youth BMI.<br>3. we considered studies that examined how changes in SSB consumption translate into changes in total energy intake and how total energy intake affects BMI.	26 (26)	Children, grades 1-12	1. School policies directed at SSB (n=5)<br>2. Availability of soft drinks and/or food in vending machines, school store/snack bar, or á la carte items at lunch (n=14)<br>3. Lunch policies (n=2)<br>4. Access and lunch policies (n=2).<br>5. Tax interventions (n=3)	Demand studies generally found that price affects soda consumption<br><br>No study found a substantial effect of soda prices on BMI
Nakhimovsky, 2016	This review compiles evidence from MICs, assessing post-tax price increases (objective 1), changes in demand for SSBs and other products, overall and by socio-economic groups (objective 2), and effects on overweight and obesity prevalence objective 3).	9 (9)	Households	Price changes and taxes	Higher SSB prices were associated with lower SSB consumption (decreases ranging from 5 to 39 kJ per person per day given 10% price increase)<br><br>Some indication that groups with lower socioeconomic status are more responsive to price changes in middle-income countries<br><br>Estimates consistent despite variations in baseline prevalences of obesity and per person per day consumption of SSBs
Powell, 2013	This study provided a systematic review of recent U.S. studies on the price elasticity of demand for sugar-sweetened beverages	21 (41)	Children and adults, aged 3 years and up	Price changes and taxes	Higher SSB prices associated with lower SSB demand<br>Mean SSB price elasticity estimate of 1.21; a tax raising price of SSBs by 20% would reduce consumption by 24%

	(SSBs), fast food and fruits and vegetables as well as the direct associations of prices/taxes with body weight outcomes.				Evidence of impact of price changes on weight outcomes mixed	
**Systematic reviews on the impact of environmental and educational interventions**						
**Authors and year**	**Aim of the review**	**Included studies (Total)**	**Population**	**Intervention**	**Main conclusion on the impact of environment**	**Main conclusion on the impact of education**
Althuis, 2013	We expanded evidence mapping methods by demonstrating their usefulness as a tool for organizing epidemiologic research on sugar-sweetened beverage (SSB) intake and health outcomes: obesity, type 2 diabetes, metabolic syndrome, and coronary heart disease/ stroke.	19 (77)	Children aged 6-79 years	3 Healthy lifestyle counseling<br>1 Beverage delivery with targeted counseling<br>1 Beverage delivery with no counseling<br>1 Unlimited SSB<br>3 Healthy lifestyle counseling<br>1 Beverage delivery with targeted counseling<br>5 Mandated consumption<br>3 Lifestyle counseling, secondary analyses<br>1 Beverage delivery with targeted counseling	Delivery of beverages resulted in reduced consumption of SSBs 2 in the intervention group	Did not report on effectiveness of educational interventions or education combined with environmental interventions (focused on evidence mapping)
Avery, 2015	To clarify hich interventions aimed at children help to reduce the consumption of SSBs and whether these interventions lead to subsequent changes in body fatness	8 (8)	Children aged 2-18 years Healthy weight. Overweight and obese	Reducing consumption of sugary drinks, interventions ≥ 6 months in duration:<br>- Health education<br>- Promotion<br>- Food environment	Modifying the school food environment can result in reduced SSB consumption among children in a cost-effective way<br>Not all studies found a reduction in BMI associated with reduced SSB consumption	Educational interventions of medium intensity (between 4 and 10 1-h sessions delivered over a period ranging between 6 weeks and 12 months) can be effective at reducing SSB consumption in children<br>Some evidence of reduced BMI, but this is

						not consistent across studies.
Gibson, 2008	This paper presents the results of a comprehensive review of the literature up to July 2008 regarding the association between sugar-containing drinks and body weight and obesity.	3 (44)	Children aged 9-18 years	1. To reduce consumption of SSD over 1 year.<br>2. Home delivery of diet soft drinks<br>to replace normal consumption<br>for 6 months<br>3. To reduce consumption of fizzy<br>drinks via education sessions, given 5 times over 1 year<br>4. 10 weeks of high-sucrose diet<br>(.60 % from SSD) or aspartame-<br>containing drinks	Home delivery of low-calorie beverages resulted in decreased SSB consumption and weight loss in intervention group<br>Changes in school availability of SSBs resulted in decreased SSB consumption, but no changes in weight	Reduced consumption of SSBs in the intervention group<br>No significant change in mean BMI
Levy, 2011	1. This paper reviews the literature on both these sets of school policies directed at SSB consumption.<br>2. we also consider how SSB consumption affects youth BMI.<br>3. we considered studies that examined how changes in SSB consumption translate into changes in total energy intake and how total energy intake affects BMI.	26 (26)	Children, grades 1-12	1. School policies directed at SSB (n=5)<br>2. Availability of soft drinks and/or food in vending machines, school store/snack bar, or á la carte items at lunch (n=14)<br>3. Lunch policies (n=2)<br>4. Access and lunch policies (n=2).<br>5. Tax interventions (n=3)	School policies that directly target the availability of SSBs in schools (e.g., vending machines, snack bars, a la carte) are associated with reduced consumption of SSBs; stricter policies appear to be more effective<br><br>It is unclear whether broad school nutrition policies (e.g., discouraging unhealthy foods in general) are effective	Cross-sectional studies found no relationship between SSB consumption and educational program.<br><br>RCTs found that SSB consumption was reduced following educational interventions, inconsistent<br>evidence for reduction in BMI

					at reducing consumption of SSBs	
Malik, 2006	This review critically examines the current evidence for an association between intake of sugar-sweetened beverages and weight gain and obesity.	2 (30)	Children aged 7-18 years	1. Weekly home delivery of noncaloric beverages for 25 weeks<br>2. Focused educational program on nutrition and drink consumption	Home delivery of low-calorie beverages resulted in decreased SSB consumption and weight reduction in intervention group	A modest reduction in SSB consumption and a reduction in prevalence of overweight/obesity in the intervention group
Malik, 2013	We conducted a systematic review and metaanalyses of prospective cohort studies and RCTs in children and adults to provide a comprehensive summary of the literature evaluating SSBs and body weight gain.	5 (32)	Children aged 8-16 years	1. Educational program (n=2)<br>2. Replacement of SSBs with noncaloric beverage (n=3)	Home delivery of SSBs resulted in significant weight reduction in intervention group Interventions need to be sustained to ensure a lasting impact	No significant effect of educational interventions on SSB consumption<br><br>No sustained effect on weight
Mazarello, 2015	This systematic review synthesizes quantitative evidence from intervention and observational (prospective cohort and XS) studies on the determinants and correlates of SSB consumption in young children (0–6 years) using the socio-ecological model.	12 (44)	Children aged 2.3-7 years	Health education/promotion<br>Food environment	Six of 12 intervention studies targeting either parents or multiple levels (child, parent, school setting) found a reduction in SSB consumption (unable to tease apart effect of food environment vs. education interventions)	Six of 12 intervention studies targeting either parents or multiple levels (child, parent, school setting) found a reduction in SSB consumption (unable to tease apart effect of environment vs. education interventions)
