# Supplementary material for: Impact of Measures Aiming to Reduce Sugars Intake in the General Population and Their Implementation in Europe: A Scoping Review
Source: Int J Public Health. 2022 Jan 13;66:1604108. doi: 10.3389/ijph.2021.1604108 (PMC8791851; doi:10.3389/ijph.2021.1604108)
Supplement: Supplementary file 3 [file DataSheet3.pdf]

Supplementary file 3

Presentation of the NOURISHING framework

The NOURISHING framework formalizes possible policies to promote a healthy diet across three domains (food environment, food system, and behavior change communication), and 10 sub-policies areas represented by the letters of the word NOURISHING.

1) Food environment:

- **N**utrition label standards and regulations on the use of claims and implied claims on food
- **O**ffer healthy food and set standards in public institutions and other specific settings
- **U**se economic tools to address food affordability and purchase incentives
- **R**estrict food advertising and other forms of commercial promotion
- **I**mprove nutritional quality of the whole food supply
- **S**et incentives and rules to create a healthy retail and food service environment

2) Food system:

- **H**arness supply chain and actions across sectors to ensure coherence with health

3) Behavior change communication:

- **I**nform people about food and nutrition through public awareness
- **N**utrition advice and counselling in healthcare settings
- **G**ive nutrition education and skills.

Hawkes C, Jewell J, Allen K. A food policy package for healthy diets and the prevention of obesity and diet-related non-communicable diseases: the NOURISHING framework. Obes Rev. (2013) 14 Suppl 2:159-168 doi:10.1111/obr.12098

https://policydatabase.wcrf.org/
